# Co‐producing knowledge of lesbian, gay, bisexual, trans and intersex (LGBTI) health‐care inequalities via rapid reviews of grey literature in 27 EU Member States

**DOI:** 10.1111/hex.12934

**Published:** 2019-06-22

**Authors:** Nigel Sherriff, Laetitia Zeeman, Nick McGlynn, Nuno Pinto, Katrin Hugendubel, Massimo Mirandola, Lorenzo Gios, Ruth Davis, Valeria Donisi, Francesco Farinella, Francesco Amaddeo, Caroline Costongs, Kath Browne, the Health4LGBTI Network

**Affiliations:** ^1^ School of Health Sciences University of Brighton Brighton UK; ^2^ Centre for Transforming Sexuality and Gender University of Brighton Brighton UK; ^3^ School of Environment and Technology University of Brighton Brighton UK; ^4^ ILGA‐Europe Brussels Belgium; ^5^ Department of Diagnostics and Public Health, Infectious Diseases Section University of Verona Verona Italy; ^6^ Department of Neuroscience, Biomedicine and Movement University of Verona Verona Italy; ^7^ EuroHealthNet Brussels Belgium; ^8^ Department of Geography Maynooth University Maynooth Ireland

**Keywords:** co‐production, Europe, health care, inequalities, intersex, LGBTI, public health, rapid review

## Abstract

**Background:**

The health inequalities experienced by lesbian, gay, bisexual, trans and intersex (LGBTI) people are well documented with several reviews of global research summarizing key inequalities. These reviews also show how the health‐care needs of LGBTI people are often poorly understood whilst suggesting that targeted initiatives to reduce inequalities should involve LGBTI people.

**Objectives:**

To determine what is known about the health‐care inequalities faced by LGBTI people? What are the barriers faced by LGBTI people whilst accessing health care, and health professionals when providing care? What examples of promising practice exist?

**Design:**

Rapid reviews of grey literature were co‐produced with LGBTI people in 27 countries followed by a thematic analysis and synthesis across all data sets. The review included grey literature from each country that might not otherwise be accessible due to language barriers.

**Main results:**

Rapid reviews showed that LGBTI people faced various inequalities and barriers whilst accessing health care. Where heterosexuality, binary gender and assumed male/female sex characteristics were upheld as the norm, and where LGBTI people differed from these norms, discrimination could result. In consultations where LGBTI people feared discrimination and did not disclose their LGBTI status, health professionals lacked the information required for appropriate assessments.

**Conclusion:**

With greater understanding of sexual orientation (LGB people), gender identity (trans people) and sex characteristics (intersex people), combined with access to contemporary knowledge and training, health professionals can work in collaboration with researchers, policymakers and LGBTI people to develop systems that are better attuned to the needs of all service users.

## 1 INTRODUCTION

The health inequalities of lesbian, gay, bisexual, trans and intersex (LGBTI) people are well documented in global research.1In this paper, we use the abbreviations LGBTI, LGBT and LGB consciously, to represent the discussion of different subsets within LGBTI in the reviewed literature. Several recent systematic reviews and narrative syntheses of research summarize these health inequalities.^1^, ^2^, ^3^ Large‐scale global reviews increasingly reflect how the health and health‐care needs of LGBTI people are often poorly understood with evidence of a higher burden of certain conditions for both the physical health and mental health of LGBTI people compared with the general population.^1^, ^2^, ^3^, ^4^

Health inequalities are documented in a range of areas including increased rates of HIV and STIs in gay, bisexual and other men who have sex with men.^1^ Also, reviews of studies on weight discrepancies in LGB people showed a higher risk of raised weight increasing sequentially with age.^5^, ^6^ LGB people reported experiencing worse physical health compared to the general population with gay men showing a higher burden of gastrointestinal problems, liver and kidney problems,^7^ and lesbian women higher rates of polycystic ovaries compared with women in general.^7^ Of LGB groups, the general health of bisexual people is poorer compared with heterosexual, lesbian and gay counterparts partly due to biphobia that exists in both heterosexual, lesbian and gay communities.^8^

International research trends suggest that LGB people are at a higher risk of developing certain types of cancer commonly diagnosed at a younger age compared with the general population,^9^, ^10^ where gay and bisexual men are twice as likely to report a diagnosis of anal cancer with those who are HIV‐positive being at the highest risk.^1^ Those LGB people who survived cancer may benefit from additional support post‐treatment to help them regain a sense of well‐being.^9^, ^11^

A review of trans health needs indicated that across global health‐care settings, trans people experienced significant health inequalities with higher rates of HIV and other STIs, mental distress, substance use and experiences of abuse (violence and discrimination) compared with non‐trans or cisgender people.^2^

In relation to mental health, research suggests that LGBT people are at higher risk of poor mental health compared to the general population with the incidence of suicidal ideation, anxiety and deliberate self‐harm markedly raised.^2^, ^4^ Gay and bisexual men showed higher rates of recreational drug use, found to be most prevalent in those aged 25‐45, and lower in those aged 45 and beyond.^4^, ^5^

Primary research exploring the health profile of intersex people is limited.^12^, ^13^ Studies undertaken often fail to account for the views of intersex people themselves, focusing instead on biomedical conditions and surgical outcomes.^12^, ^14^ Further research is needed in collaboration with intersex people to understand their experiences of accessing health care.^15^ The same applies to research with trans and LGB groups, where much scope remains to include LGBTI people in research. Collaborative research with LGBTI people could inform future service delivery.^16^

### 1.1 Co‐production

The above‐mentioned global reviews are helpful as they provide an overview of health inequalities in terms of ‘what is known’ and where further research is needed; however, some studies are based on research that is done about LGBTI people instead of being undertaken in partnership with them. Research communities commonly regard primary research with robust quantitative designs as most rigorous,^17^ or systematic reviews, meta‐analyses or meta‐syntheses as most useful in reflecting global trends for a specific field across data.^1^ However, rich and more nuanced information can be contained in grey literature representing service user experiences and views. Patient (or service user) and Public Involvement and Engagement (PPIE) in research and health service provision has grown significantly since confirmation of the World Health Organization Alma‐Ata Declaration that marked the start of an international commitment to making health care equally accessible to all.^18^ The principles underpinning PPIE include actively involving service users in research and the organizations that conduct research, and involving service users in sharing knowledge of the research with the public.^19^

This is essential action as global evidence mounts that LGBTI health inequalities do not necessarily stem from individual behaviour, genetic factors or lifestyle factors. Instead, some LGBTI people may encounter discrimination based on their sexual orientation, gender identity and sex characteristics.^20^, ^21^, ^22^ A review of LGBTI health‐care inequalities found when people access health care, they may experience minority stress associated with sexual orientation and gender identity,^5^, ^23^ heteronormativity (cultural and social norms that preference and prioritize heterosexuality),^24^, ^25^ victimization^26^ and discrimination combined with the effects of stigma.^27^, ^28^, ^29^ Furthermore, in global settings where LGBTI people were not legally protected against discrimination, they were more apprehensive when accessing health care due to anticipated or internalized stigma where they devalued themselves that may cause barriers in communication between LGBTI people and health professionals.^7^, ^8^, ^27^ These factors such as discrimination and minority stress are linked to the causes of health inequalities; however, the causes are complex and often a combination of a range of individual as well as cultural, political and social factors.^5^, ^7^, ^13^ Efforts to reduce LGBT health and health‐care inequalities is a social justice issue requiring targeted research, policy and practice intervention at multiple levels.^3^, ^30^ Consequently, research with LGBTI people and their engagement in health service delivery, research and policy is increasingly important as a collaborative effort to tackle inequalities.^3^, ^16^, ^31^ LGBTI people should be included in decision making to represent their specific health concerns, and by helping to develop progressive services.^3^, ^31^, ^32^

Along these lines, the principles of involvement and engagement were maintained in a European study entitled Health4LGBTI carried out by a Consortium of five EU partners appointed by the European Commission and funded by the European Parliament. The Consortium consisted of academic institutions, a Public Health body and key stakeholder associations.

The Health4LGBTI study was organized according to five thematic areas, each of which was managed according to a co‐partnership arrangement involving a pairing of two of the Consortium partners.^13^, ^20^, ^33^, ^34^, ^35^ The LGBTI stakeholder association ILGA‐Europe was a co‐partner on all the research and communication activities to ensure that the overall Health4LGBTI study was designed and carried out with and by members of LGBTI communities (instead of about them). Furthermore, LGBT people were represented within the research teams of all the partners and in the project advisory board.

Co‐production was understood as a considered process where LGBT people were actively and meaningfully involved in every aspect of the research: as co‐applicants on the funding application; by helping to identify the research priorities; by contributing to planning, designing and implementing the research process; and by participating in the debate at policy level about the practical application of the results of the study.

**Figure 1:**
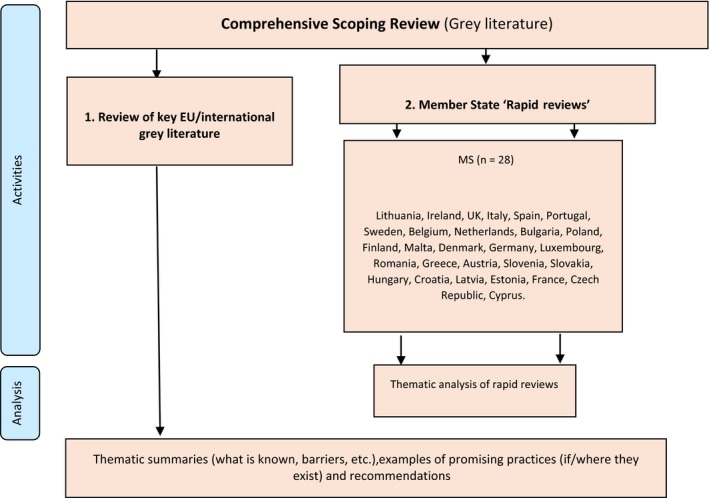
Overview of the Comprehensive Scoping Review (CSR)

In particular, the partnership with ILGA‐Europe enabled access to other key advocacy groups such as Oii (Organisation Intersex International) and to grassroots NGOs in all the European Union Member States who played a key role in conducting the comprehensive scoping review (CSR; Figure 1).

## 2 OBJECTIVES

The CSR was centred around the following core questions that were developed with ILGA‐Europe and LGBT people who formed part of the consortium's research teams: (a) What is known about the health‐care inequalities faced by LGBTI people?; (b) What are the potential barriers faced by (i) LGBTI people when using or trying to access health care and (ii) health professionals when providing care for LGBTI people?; and (c) What examples of promising practice exist to address the specific health needs of LGBTI people?

## 3 METHODS

A critical realist framework was used to explore the research questions via a collaborative and accessible methodology.^36^ The review followed a participatory approach where knowledge was co‐produced between researchers, ILGA‐Europe and LGBTI representative in each country by means of an adapted version of the Arksey and O'Malley's^37^ framework for scoping studies. The framework guided every stage from identifying the question, identifying the relevant literature, LGBTI experts selecting the literature in their own countries, charting the data according to an overarching thematic analysis, reporting and consulting stakeholders for feedback (Table 1).

**Table 1:** Framework for conducting scoping studies (adapted from Arksey and O'Malley, 2005)

Stage	Description
1. Identifying the question	Identifying the research question provides the roadmap for subsequent stages. Research questions are broad in nature as they seek to provide breadth of coverage
2. Identifying relevant studies or literature	Identifying relevant studies and developing a decision plan for where to search, which terms to use, which sources are to be searched, time span, and language(s). Example sources include electronic databases, reference lists, hand searching of organisations and relevant conferences. Although breadth and practicalities of the search are important, clear parameters should be made upfront about how these will impact the search criteria (inclusion/exclusion)
3. Study or literature selection	Literature selection involves *post hoc* inclusion and exclusion criteria. These criteria are based on the specifics of the research question and on new familiarity with the subject matter through reading the studies and/or literature
4. Charting the data	A data‐charting form is developed and used to extract data from each study. A 'narrative review' or 'descriptive analytical' method is used to extract contextual or process oriented information from each study
5. Collating, summarising, and reporting results	An analytic framework or thematic construction is used to provide an overview of the breadth of the literature. A thematic analysis is then presented
6. Consultation	Opportunities for stakeholder involvement (eg advisory board peer review)

The CSR included two tasks (see Figure 1): (a) a review of key European/international grey literature (policies, guidelines and legislation) and (b) rapid reviews of relevant grey literature from European Member States that may not be accessible due to language barriers. This paper presents findings of rapid reviews of relevant grey literature from 27 countries. A comprehensive overview of policy, guidelines and legislation is not included here due to the volume of data generated via the rapid reviews.^33^ Partner organizations of ILGA‐Europe identified LGBTI experts in each European Member State to conduct ‘rapid‐reviews’ of relevant grey literature from their own countries. These LGBTI contacts were involved in every stage from designing the template, identifying the literature and summarizing content for their country. The aim was to access grey literature that might not otherwise be accessible (eg non‐English and/or not indexed in scientific databases), ensuring a good geographical coverage of the information and data collected by embracing different social and cultural contexts.

### 3.1 Inclusion criteria

Inclusion of key EU/international grey literature in rapid reviews was determined by focusing on the core objectives. Literature that was published by relevant institutions and national organizations but not indexed in scientific databases was included. Some geographical restrictions were applied by preferencing grey literature relating to single European Member States. Other inclusion criteria were language (published in English or translated to English) and timeframe (2006‐2016). Rapid reviews explored a number of indicative areas as set out in Table 2.

**Table 2:** Inclusion/exclusion criteria for rapid reviews of grey literature

Inclusion	Exclusion
Literature focusing on the Comprehensive Scoping Review core questions and published by relevant institutions and international or national organizations	Academic/scientific literature/grey literature focusing on LGBTI lives and general concerns
Grey literature relating to a single European Member State	Literature relating to multiple countries or European Member States
Grey literature including 1) research and/or evaluation studies (eg questionnaires and surveys, and interviews) not published in academic journals, on perceived or experienced discrimination by LGBTI people regarding health care; 2) relevant MS guidance, frameworks, policies and/or legislation referring specifically to LGBTI people and health care (eg these could be local, regional or national policies/legislation); 3) complaint information or data concerning perceived or experienced discrimination by LGBTI people relating to health care; and 4) examples of promising practices which engage with LGBTI people regarding access to health care (eg descriptions of projects, programmes, initiatives, policies, working practices and procedures)	Scientific articles published in formal peer‐reviewed journals or other forms of academic publishing and distribution channels
Published between 2006 and 2016	Prior to 2006
Published in English or translated to English	Non‐English or not translated to English

### 3.2 Data extraction and synthesis

Information from Member States was gathered via a rapid‐review template (see Appendix S1) designed specifically for the purposes of the Health4LGBTI project with LGBTI people, GLEN (Gay and Lesbian Equalities Network) and the Estonian LGBT Association. A pilot was undertaken in Ireland and Estonia during April 2016, to test the efficacy of the template prior to commencing the review process in all EU Member States. Following minor revisions, the template was sent out to the remaining Member States for completion between May 2016 and August 2016.

Of the 28 EU Member States consulted, contacts in 27 countries completed the template for each document they reviewed except for Cyprus. Most reviews were completed in English; however, data presented via rapid reviews were the work of LGBTI contributors from specific countries, which meant some reviews translated summaries of texts only available in national languages. These reviews were translated to English. This is a key strength of this scoping review in being able to access literature that might otherwise be ‘hidden’. The review processes utilized were not designed to evaluate the quality of grey literature but instead scope available literature. Data sets for each country varied in scope with reviews summarizing between 4 (Luxembourg) to a maximum of 40 (Germany) pieces of grey literature.

Each of the returned rapid reviews was edited for consistency and accessibility in terms of language and structure followed by a thematic analysis.^38^, ^39^ Themes were recorded in an Excel spreadsheet, coded and marked where they recurred for each country. Codes were reviewed and agreed between two analysts, and themes that did not have enough data to support them were discarded along with themes that did not address the research questions.^38^ The process of editing, thematic analysis and coding was co‐produced between the first two authors. The scientific review of literature undertaken before the comprehensive scoping review^13^ provided the theoretical framework for the analysis. The results that follow present examples of overarching themes that were developed to reflect the content across all 27 rapid‐reviews.

## 4 RESULTS

Since LGBTI health inequalities were reported elsewhere in a review of global peer‐reviewed literature,^3^ results in this paper cover recurring themes identified across rapid reviews according to the following questions:

### 4.1 What are the potential barriers faced by LGBTI people when accessing health care?

Rapid reviews revealed a range of barriers faced by LGBTI people when accessing health care. Themes that recurred across data sets were as follows: heteronormativity and gender normativity, prejudicial attitudes of health professionals (eg signposting to conversion therapy); fear of coming out and revealing their LGBT status; and the unnecessary medicalization of intersex variance. The quotes that follow are specific to each country and in some instances divergent views were noted; however, quotes are useful to illustrate themes. Only the themes that recurred across a number of data sets were included (see Tables 3 and 4).

**Table 3:**
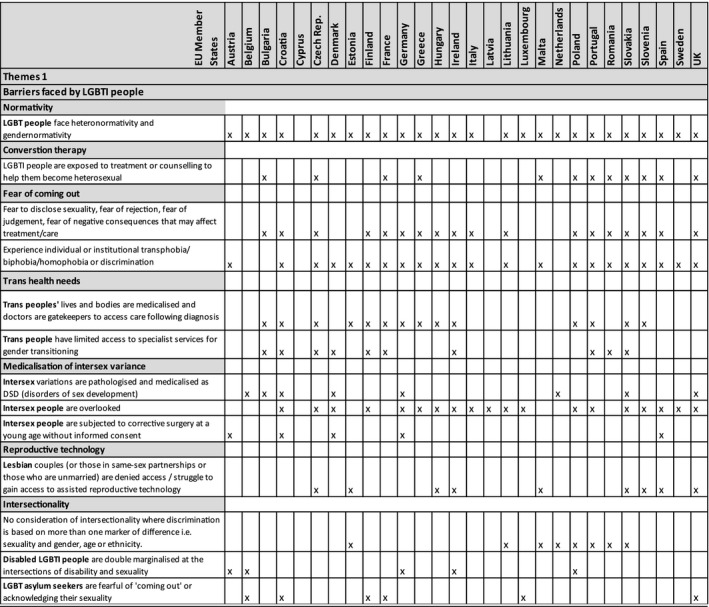
Thematic analysis 1

**Table 4:**
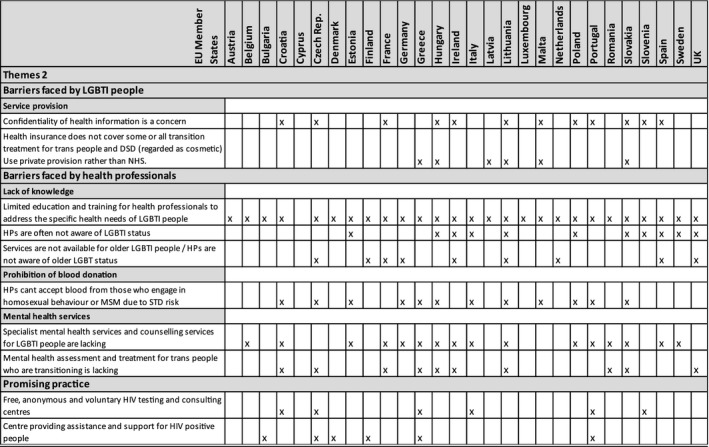
Thematic analysis 2

#### 4.1.1 Theme: Normativity

Heteronormativity and gender normativity were visible in most rapid reviews submitted by Member States [x26 MS]. This occurs where gendered norms of masculinities/femininities are upheld, or where heterosexuality is sustained as the status quo. The lives and bodies of LGBTI people seemingly disrupt such dominant norms of sex, sexuality and gender. Lesbian, gay, bisexual, trans and intersex people were reported as being treated as ‘other’ leading to marginalization.

My doctor told me a couple of times that I do not fulfil the requirements of the looks. She wanted me to change my looks and the way I behave. She told me that my hair is wrong, my clothes are not good, despite the fact that I wear rather neutral clothing. She told me that the transition is not about my happiness, but about how I fit into society. (Trans person, 24 year‐old, Slovakia)

(Source: Guidebook ‐ Transfúzia 2015 The standards of trans‐inclusive environment in the healthcare system. Transfúzia)

The Slovakian rapid review reflects how a health professional attempt to help a trans person change their gender expression to conform to gender norms related to clothing, behaviour and hairstyle that follow traditional representations of masculinities/femininities. Some trans people do not fit normative categories as their gender expression disrupts commonly accepted ideals. An Austrian paper for psychotherapists suggests that health professionals should remain open to plurality in gender expression and include those who differ from the norm:

…some of her colleagues are not willing to get in touch with the life of other (i.e. trans) people, for example when those psychotherapists talk about their clients like ‘he really looks like a woman’ or ‘she thinks she is a man’. Psychotherapists who cling to normative categories should not provide psychotherapy. (Austria)

(Source: Magazine Article – Kunert, C. 2014 What’s the point of that masquerade? WLP News, Zeitschrift des Wiener Landesverbandes für Psychotherapie)

Rapid reviews indicated where health practitioners show limited awareness of the impact of upholding traditional gendered norms, training and greater awareness of diversity and plurality of gender, sex and sexuality would aid LGBTI people to access care without experiencing judgement.

#### 4.1.2 Theme: ‘Conversion therapy’

Data from the rapid reviews suggest that the widely condemned practice of ‘conversion therapy’ persists in some European Member States [x12 MS: Bulgaria, Czech Rep., France, Greece, Malta, Poland, Portugal, Romania, Slovakia, Slovenia, Spain, UK]. ‘Conversion therapy’ is based on assumptions that homosexuality, bisexuality and/or trans identities are a mental disorder, or questionable based on religious beliefs and should be ‘cured’ as seen in these quotes:

The psychologist that I visited the last time is religious – she is a Christian. I am too, but not so much… When I opened up to her regarding my sexual orientation, she agreed to counsel me but only if I agreed to change my life and my orientation. She tried to send me to [name of a pilgrimage site], told me that they will cure me there of this compulsion… But I don’t want to give this up, I was very sad. (Quote from survey with LGBT people, Slovakia)

(Source: Guidebook ‐ Smitková & Kuruc, 2012 Recommendations and incentives for psychologists working with lesbian, gay, bisexual and trans (LGBT) clients. Iniciatíva Inakosť)

Even though ‘conversion therapy’ as a practice still appears to exist in some MS, a welcome diversion to this practice is evident in Malta and the UK where the law is under review to prohibit conversion therapy and thereby protect LGBT people and vulnerable minors.

(Source: Frye, 2016; TGEU, 2016; UK LGBT Action Plan 2018)

#### 4.1.3 Theme: Fear of coming out

Several MS rapid reviews reported on grey literature that showed how some LGBTI people feared ‘coming out’ to their peers, health professionals and in social settings due to potential negative consequences [x18 MS: Bulgaria, Croatia, Czech Republic, Finland, France, Germany, Greece, Hungary, Ireland, Italy, Lithuania, Poland, Portugal, Romania, Slovakia, Slovenia, Spain, UK]. Data from a Lithuanian information brochure highlighted the experience of a lesbian woman:

After experiencing the first symptoms of an illness, I feel huge emotional stress, because I know that after turning to a healthcare facility either I will have to ‘come‐out’ as lesbian and to shock my doctor or I will have to conceal this fact and to face many misguided questions. As long as I have the choice, I will stay at home and will try to treat myself independently. The healthcare sector is not understanding my needs. (Lesbian woman, Lithuania)

(Source: Brochure – LGL 2010 Ten things about LBT women’s health. The National LGBT* Rights Association)

What became clear from the rapid reviews was, that based on sexual orientation and gender identity, LGBT people were commonly treated differently in health systems with some fearing negative consequences.

#### 4.1.4 Theme: Medicalization of intersex variance

Eight rapid reviews mentioned intersex people and concerns over unnecessary pathologization and medicalization where intersex variations are regarded as ‘disorders of sex development’ or ‘DSD’ within biomedicine and their related systems of diagnosis and classification. However, the term DSD is highly contested by intersex people themselves [x8 MS: Belgium, Bulgaria, Croatia, Denmark, Germany, Netherlands, Slovakia, UK].

The medical healthcare still has the monopoly on knowledge on inter*conditions. ‘DSD’ is taboo and gets too little attention in healthcare settings. Medical teams need to work more multidisciplinary and need to be aware of the nonsense of binary thinking (male/female). Professionals within healthcare don’t have the right education to deal with inter*. Research about the needs of inter*people is focused on medical issues rather than sociological. (Doctor, Belgium)

(Source: Presentation – Cools, M. 2013 Inter*, an introduction: Body and gender: past simplicity. UZ Ghent)

As the bodies and sex characteristics of some intersex people do not fit the male/female binary, intersex people may be subjected to normalizing surgery at a young age without informed consent [x5 MS: Austria, Croatia, Denmark, Germany, Spain].

While intersex children may face several problems, in the ‘developed world’ the most pressing is the ongoing Intersex Genital Mutilation, which present a distinct and unique issue constituting significant human rights violations. (Austria)

(Source: NGO Report – VIMO 2015 Intersex genital mutilation. Human rights violations of persons with variations of sex anatomy. Verein Intersexueller Menschen Österreich (VIMÖ) & Zwischengeschlecht.org)

The rapid review from Austria represented normalizing surgery of intersex minors as harmful, whereas the rapid review from Germany highlighted literature stating that surgery on intersex minors with variance in sex characteristics to align their body with male/female sex markers can be regarded as interference with the right to physical integrity and bodily autonomy. Decisions that impact on the physical integrity of intersex people should be based on their right to self‐determine and any intervention should occur in the context of informed consent.

### 4.2 What are the potential barriers faced by health professionals when providing care for LGBTI people?

Rapid reviews identified barriers health professionals may face when providing care for LGBTI people such as lack of knowledge concerning the lives and health‐care needs of LGBTI people; lack of awareness or consideration of the sexual orientation, gender identity or sex characteristics of LGBTI people who access health services; limitations around the prohibition of blood donation; or a lack of specialist mental health services and counselling services for LGBTI people.

#### 4.2.1 Theme: Lack of knowledge

All rapid reviews specifically drew attention to literature highlighting the seemingly limited education and training opportunities available for health professionals to address the specific health needs of LGBTI people in Member States [x27 MS].

Early on in my smear history I told a nurse that I had a female partner and she was completely taken back and said ‘I don’t know what to do about that’… she was really confused as to what to do next clinically… she said ‘well you are here and we can do it anyway’ but she hadn’t been trained for that situation (Lesbian woman, UK)

(Source: Report – Bottomley et al., 2014 Are you ready for your screen test? The Lesbian and Gay Foundation & University of Salford)

Examples from the grey literature showing the need to increase knowledge to tackle ignorance around LGBTI issues both in the negative statements and viewpoints, and in the quote below the more positive (eg self‐reflection and recognition by health professionals that they need specific training for example to support LGB youth who might be struggling):

I think you have to be very precise and I personally think that I do not have sufficient knowledge, information, ideas on how to deal with it. How to guide a young person who is in an identity crisis? What am I? Am I gay, lesbian, bisexual? What does that mean? How do I bring it out or how do I do that? (Health professional, Netherlands)

(Source: Report – Emmen et al., 2014 Jong & Anders. Nederlands Jeugdinstituut en Movisie)

#### 4.2.2 Theme: Prohibition of blood donation

A number of rapid reviews drew attention to literature demonstrating examples where some health professionals were prevented from accepting blood donated by those who engaged in same‐sex sexual behaviour or men who had sex with men, due to the perceived risk of sexually transmitted infections [x12 MS: Croatia, Czech Republic, Estonia, Germany, Greece, Hungary, Italy, Lithuania, Malta, Poland Portugal, Slovakia].

Gay and bisexual men are often excluded (from) blood donation, although this exclusion is not required nor allowed by law. The law only requires the permanent exclusion of people ‘whose behaviour exposes them to high risk of acquisition of STIs’, or the temporary exclusion (4 months) of all those people ‘who have occasional sex’. The law never mentions homosexuals or men who have sex with men. However, LGBTI organisations are often informed about cases of permanent exclusion after direct questions about sexual orientation. (Italy)

(Source: Law – Italian Ministry of Health 2005 Decreto del ministro della salute 3 marzo 2005 “Protocolli per l'accertamento della idoneità del donatore di sangue e di emocomponenti”)

Even where the exclusion of MSM did not exist as a legal requirement, people may have been turned away by health professionals as gatekeepers to these services. Across the rapid reviews, data suggested that many LGBTI people anticipated negative consequences when disclosing their sexual orientation, gender identity or sex characteristics to health professionals. Moreover, it also seems that some health professionals have a limited awareness of equal rights and the protected nature of sexual orientation and gender identity in many European Union Member States.

#### 4.2.3 Theme: Lack of mental health services

Due to multiple layers of marginalization, many LGBTI people may experience discrimination and stigmatization. Consequently, the incidence of mental health problems can be much higher for this population compared with the general population. However, much grey literature reported by Member States highlighted how specialist mental health services and counselling services for LGBTI people are generally lacking [x16 MS: Belgium, Croatia, Estonia, France, Germany, Greece, Hungary, Ireland, Italy, Lithuania, Poland, Portugal, Romania, Slovakia, Spain, Sweden].

### 4.3 What examples of promising practice exist to address the specific health needs of LGBTI people in your country?

The rapid review template requested examples of promising practice in addressing the specific health needs of LGBTI people in EU countries. Examples provided spanned a broad range of settings such as HIV testing and support centres where free, anonymous and voluntary HIV testing and consulting centres were provided [x5 MS: Croatia, Czech Republic, Italy, Portugal, Slovenia]; centres providing assistance and support for people living with HIV [x6 MS: Bulgaria, Czech Republic, Denmark, Finland, Greece, Portugal]; peer mentoring for LGBT people in crises (Czech Republic); a queer social group to interact with refugees and thereby foster mutual understanding (Luxembourg); information leaflets for health professionals to address LGBTI health (Poland); queer leadership development, counselling and psychological support (Slovakia); and a suicide prevention strategy for LGBT people (Italy).

## 5 DISCUSSION

The results of the rapid reviews consistently demonstrated a range of health‐care inequalities, barriers to accessing and providing care, and discrimination based on gender identity, gender expression, sexual orientation and sex characteristics for LGBTI people. Some LGBTI people feared negative consequences such as being treated as different or as ‘other’ whilst accessing (or attempting to access) health care.^11^, ^40^ Due to the effects of discrimination and stigma, research reported that specialist mental health or psychological support services for LGBTI people where they could make meaning of adversity were lacking.^2^, ^3^, ^4^, ^11^, ^12^ Rapid reviews were consistent with wider academic literature in reporting that gay, bisexual and trans people can be deterred from accessing health care such as seeking HIV testing and treatment if they feared discrimination or encountering the stigmatizing attitudes of health professionals.^27^ The reviews reported literature stating that LGBTI people were either prohibited from donating blood where they had engaged in same‐sex sexual practices, or another example where they were signposted to conversion therapy as a treatment option to help ‘cure’ them. In relation to conversion therapy, health professionals’ assumptions framing LGBT identities as ‘disorders’ were based on dated diagnoses that were removed from the psychiatric systems of diagnosis and classification (DSM and ICD) as part of the demedicalization of sexual orientation.^41^ This lack in knowledge supports the need for education and training of health professionals widely reported in research to question normativity and promote more inclusive health‐care practices for LGBT people.^1^, ^2^, ^3^, ^4^, ^24^ Health professionals will benefit from further education and training to help them navigate their way through changing terminology and complex health‐care systems. For example, even though sexual orientation was demedicalized, the classification of gender dysphoria that frames trans people as gender non‐conforming persists in the DSM‐5.^42^ Whilst these categories unnecessarily label trans people, the diagnosis acts as a gateway to hormonal treatment, surgery and the related medical technologies many trans people require to align their bodies and gender identity.^43^

Similar restrictions based on biomedical diagnoses of intersex people apply. Intersex relates to a range of physical traits or variation that lie between binary ideals of male and female where many forms of intersex variance exist, whilst understanding sex as a spectrum rather than a binary category.^14^, ^15^, ^20^ A range of intersex variations are diagnosed as ‘disorders of sex development’ (DSD) which unnecessarily medicalize intersex people based on physical difference. These diagnoses can be incongruous with how intersex people self‐identify. Much of the research on intersex health relates to surgical intervention that is focused on assigning one sex within the male/female binary often without consent in relation to intersex minors. More research is needed to account for the views of intersex people themselves regarding their health and experiences of accessing health care.^3^, ^12^, ^15^, ^44^

Notwithstanding the value of and limitation associated with biomedical classification, the Yogyakarta Principles guide to human rights affirm binding international legal standards regarding LGBT people where ‘Everyone has the right to the highest attainable standard of physical and mental health, without discrimination on the basis of sexual orientation or gender identity’ (Principle 17).^45^ Through changes in legislation, significant progress has been made towards achieving equality for LGBT people in Europe^21^, ^22^, ^46^ and the UK.^47^ Awareness of the need to assert the rights of LGBTI people is increasing with the knowledge of protection against discrimination based on sexual orientation (lesbian, gay, bisexual people), gender identity (trans people) and sex characteristics (intersex people).^3^ As the struggle for recognition of LGBTI people's fundamental rights persists, LGBTI activists, NGOs, researchers and practitioners are working in collaboration to campaign for full recognition including legal recognition of gender, non‐discrimination in the workplace, non‐discrimination when accessing services provided by public‐facing organizations, and freedom of expression.^46^, ^47^

Health inequalities can be better tackled where normativities in relation to gender, sexuality and sex characteristics are questioned. Heteronormativity implies that people’s gender and sex are by nature and align with opposite‐sex attraction as the only conceivable way of being ‘normal’.^24^, ^40^, ^48^, ^49^ Rapid reviews showed how health‐care inequalities occur in contexts of heteronormativity where heterosexuality is upheld as a key social and cultural norm. Broader research shows in health‐care settings where LGBTI people access care, being heterosexual is often assumed as a given.^24^, ^25^ LGBTI people are marginalized due to heteronormative or gender normative assumptions conveyed in communication between health professionals and their patients where language is infused with subtle meaning.^25^, ^49^ These assumptions are heard in verbal communication and seen in written communication where case notes and multidisciplinary forms often fail to recognize the lives and partnerships of LGBTI people.^50^, ^51^ The actions of health professionals may be (un)intentionally insensitive towards LGBTI people.^25^, ^40^ When LGBTI people are overlooked due to assumed heterosexuality, cisgenderism (non‐trans) and normative sex characteristics (intersex), the relationship between health providers and people who access care is adversely affected. In these instances, LGBTI people who access health care and other support services are less likely to be open and disclose their sexual orientation, gender identities or sex characteristics in the first few consultations, or they may be hesitant to share information relevant to their specific needs.^24^, ^25^, ^52^ Consequently, health professionals may not have all the relevant information needed to make adequate assessment of their health needs when suggesting appropriate treatment options.^53^, ^54^ However, research highlights where health‐care practitioners did acknowledge the sexual orientation of service users or made visible their own sexual orientation, these encounters fostered open and inclusive communication whilst respecting sexual and gender plurality.^16^, ^32^, ^49^, ^51^

## 6 CONCLUSION

Training could help health professionals understand the lives and historic events that may have marginalized LGBTI people, to improve access to safe and supportive practice that are sensitive to the fear or anxieties of LGBTI people when accessing care. Further scope remains to include this kind of information in undergraduate curricula for medical students, nursing students and those studying allied health professions. With appropriate skills and training, all health‐care workers can aid open communication with LGBTI people, where practitioners use inclusive and non‐normative language by avoiding assumed heterosexuality or binary gender. When opportunities are created for LGBTI people to disclose their identity in communication with health professionals that upholds values of mutual respect, or the visibility of LGBTI staff is promoted when working with LGBTI patients, these measures may help to create an atmosphere where people feel more comfortable to access care and discuss their specific health needs. Although some encouraging promising practices were evident, there is nevertheless still much to be done to ensure that the fundamental rights of LGBTI people are honoured. Promising practices included LGBTI people accessing HIV testing and consulting services where their confidentiality and anonymity were respected or gaining access to psychological services provided via peer‐to‐peer support mechanisms. The challenge for health professionals who work in collaboration with LGBTI people is to develop the structures for general and specialist health‐care provision that are truly inclusive and equally accessible to all regardless of gender identity, sexual orientation or sex characteristics. Appropriate training for health professionals, co‐facilitated by LGBTI people across all health systems, is an important step in this direction.

## 7 LIMITATIONS

Data presented via rapid reviews were the work of LGBTI contributors from 27 countries which meant some reviews translated summaries of texts only available in national languages. Whilst this is a key strength of this scoping review in being able to access literature that might otherwise be ‘hidden’, it also means that the authors were unable to verify the appraisals of literature or accuracy of translations. The processes utilized in this rapid review were not designed to evaluate the quality of grey literature but instead scope available literature in each EU country.

The rapid‐review protocol asked for LGBTI experts to differentiate (where possible) between L, G, B, T and I people when reporting on literature from their countries. However, in some cases it is unclear which group(s) the literature reported was referring to. Consequently, where this was unknown, the full acronym of LGBTI was used.

## CONFLICT OF INTEREST

None declared.

## ETHICAL APPROVAL

Ethical approval for the CSR was not needed as the study did not collect any primary data. Other components of the study such as focus groups and training gained ethical approval in Poland and Italy as well as via the College Research Ethics committee (CREC) at the University of Brighton.

## DATA ACCESSIBILITY

The data that support the findings of this study are available from the corresponding author upon reasonable request.
