# Supplementary material for: Co‐producing knowledge of lesbian, gay, bisexual, trans and intersex (LGBTI) health‐care inequalities via rapid reviews of grey literature in 27 EU Member States
Source: Health Expect. 2019 Jun 22;22(4):688–700. doi: 10.1111/hex.12934 (PMC6737757; doi:10.1111/hex.12934)
Supplement: Supplementary file 1 [file HEX-22-688-s001.docx]

**Supplementary file**

| **What is known about the inequalities faced by LGBTI people as it relates to healthcare settings? (e.g. mental health, sexual health, general health)** | |
| --- | --- |
| **About the document/grey literature identified** | **Description/explanation** |
| Title of document: |  |
| Authors: |  |
| Publisher/produced by: |  |
| Date published/produced: |  |
| Type of document (e.g. report, leaflet, flyer, evaluation, complaints data, training module, policy documents, guidance etc.) |  |
| What level is the document referring to? (local/regional/national) |  |
| Target group(s) – L.G.B.T.I – please be precise |  |
| Target group(s) – If relevant, which vulnerable LGBTI sub-populations are referred to? (e.g. young, elderly, refugees, immigrants, disabled, socially isolated, those in poverty etc.) |  |
| Document available in a national language or English? |  |
| Website link to the document (if in English) |  |
| **Content of the document/grey literature** |  |
| Please provide a **short summary** of the document in English (e.g. max 300 words). If a study, please try and include the main aim of the study, methods used (e.g. interviews, survey), sample size, LGBTI profile, which health issue, main findings, and conclusions. |  |
| What are the **key healthcare inequalities** identified by the document? |  |
| What **type of data** (if any) is presented (e.g. from a questionnaire, survey, interviews, complaints data etc.)? |  |
| What kind of **healthcare setting** is referred to? (e.g. GP, hospital, private clinic, mental health etc.) |  |
| What kind of **healthcare worker** is referred to (e.g. nurses, clinicians, psychologists, surgeons etc.)? |  |
| Are there any **key quotes, statements, or examples** provided in the document that might be useful as ‘stimulus’ or discussion material in a **training module** for healthcare professionals on LGBTI issues, inequalities and access to healthcare health professionals on LGBTI access to healthcare? |  |
| **Anything else to add?** |  |
| Any other comment? |  |

Appendix. Rapid-review template
